# Forensic mental health in Europe: some key figures

**DOI:** 10.1007/s00127-020-01909-6

**Published:** 2020-07-10

**Authors:** Jack Tomlin, Ilaria Lega, Peter Braun, Harry G. Kennedy, Vicente Tort Herrando, Ricardo Barroso, Luca Castelletti, Fiorino Mirabella, Franco Scarpa, Birgit Völlm, the experts of COST Action IS1302, Thierry Pham, Rüdiger Müller-Isberner, Maris Taube, Gianfranco Rivellini, Valeria Calevro, Raffaello Liardo, Michele Pennino, Inga Markiewicz, Fernando Barbosa, Erik Bulten, Lindsay Thomson, Miran Pustoslemšek, Jose Manuel Arroyo, Allan Seppänen, Florence Thibaut, Dragica Kozaric-Kovacic, Tija Zarkovic Palijan, Silvana Markovska-Simoska, Marija Raleva, Aldona Šileikaitė, Arunas Germanavicius, Ilona Čėsnienė

**Affiliations:** 1 Department of Forensic Psychiatry, University of Rostock, Gehlsheimer Straße 20, 18147 Rostock, Germany; 2 Woman, Child and Adolescent Health Unit, National Center for Disease Prevention and Health Promotion, Italian National Institute of Health, Rome, Italy; 3 Pompe Foundation Department LFPC, Forensic Psychiatric Hospital, Zeeland, The Netherlands; 4 National Forensic Mental Health Service, Central Mental Hospital, Dundrum, Ireland; 5 Department of Psychiatry, Trinity College Dublin, Dublin, Ireland; 6 Unitat Polivalent de Psiquaitria Quatre Camins, Penitentiary Psychiatry, Parc Sanitari Sant Joan de Deu, Sant Boi de Llobregat, Barcelona Spain; 7 Department of Education and Psychology, University of Tras-Os-Montes and Alto Douro, Vila Real, Portugal; 8 Azuenda Ospedaliera Carlo Poma, Matov, Italy; 9 Center for Behavioural Sciences and Mental Health, Italian National Institute of Health, Rome, Italy; 10 Department of Forensic Services, USL Toscana Centro, Florence, Italy

**Keywords:** Forensic mental health, Prevalence, Beds, Deinstitutionalization, Europe

## Abstract

**Purpose:**

While the number of forensic beds and the duration of psychiatric forensic psychiatric treatment have increased in several European Union (EU) states, this is not observed in others. Patient demographics, average lengths of stay and legal frameworks also differ substantially. The lack of basic epidemiological information on forensic patients and of shared indicators on forensic care within Europe is an obstacle to comparative research. The reasons for such variation are not well understood.

**Methods:**

Experts from seventeen EU states submitted data on forensic bed prevalence rates, gender distributions and average length of stay in forensic in-patient facilities. Average length of stay and bed prevalence rates were examined for associations with country-level variables including Gross Domestic Product (GDP), expenditure on healthcare, prison population, general psychiatric bed prevalence rates and democracy index scores.

**Results:**

The data demonstrated substantial differences between states. Average length of stay was approximately ten times greater in the Netherlands than Slovenia. In England and Wales, 18% of patients were female compared to 5% in Slovenia. There was a 17-fold difference in forensic bed rates per 100,000 between the Netherlands and Spain. Exploratory analyses suggested average length of stay was associated with GDP, expenditure on healthcare and democracy index scores.

**Conclusion:**

The data presented in this study represent the most recent overview of key epidemiological data in forensic services across seventeen EU states. However, systematically collected epidemiological data of good quality remain elusive in forensic psychiatry. States need to develop common definitions and recording practices and contribute to a publicly available database of such epidemiological indicators.

**Electronic supplementary material:**

The online version of this article (10.1007/s00127-020-01909-6) contains supplementary material, which is available to authorized users.

## Introduction

Care provided for mentally disordered offenders in the context of forensic psychiatric services varies substantially across European Union (EU) states [1, 2]. Epidemiological and service-level differences exist concerning the number of forensic beds, average length of stay, the availability of dedicated long-stay services, the proportion of male and female patients and the stratification of hospitals into different security levels [1, 3]. Differences are also observed regarding legal frameworks stipulating under what circumstances individuals are to be admitted into secure care and what this care may look like [2, 4].

The literature describes some of these differences. Chow and Priebe [3] have reported that in 2011 forensic bed rates per 100,000 varied from approximately 1 (Switzerland) to 12 beds (the Netherlands) per 100,000. A decade earlier in 2002 a similar gap was observed between Portugal with 2.2 and Sweden with 10.4 beds per 100,000 [1]. Average lengths of stay differ between states. Data collected between 2013 and 2014 indicate that average length of stay varied from 4 years in Italy to 10 years in Belgium, England, the Netherlands and Serbia [2]. The proportion of male and female patients is also heterogenous. Salize and Dreßing [1] report that in the Netherlands in the year 2000, 5.1% of patients were female; in Austria in 2001, this was 16.3%.

This variation in service provision is not unique to forensic services [5]. Becker and Kilian [6] highlight differences across general mental health services in Europe. Recently, Sadeniemi et al. [7] described the differences in service provision between regions in Finland and Spain. They report ‘… 2.5 times more physicians, 4.5 times more psychologists, and 15.1 times more psychiatric nurses…’ in the former than in the latter. This is despite findings that the prevalence of mental illness in these two countries is approximately the same as it is for other European states [8]. Further, Dreßing and Salize [9] reported that rates of compulsory admission in a sample of European countries varied from 6 (Portugal) to 218 (Finland) per 100,000.

However, the reasons for these disparities are not well understood. Becker and Killian [6] propose that in general mental health settings heterogeneity in service provision is in part attributable to economic, political and sociocultural variation. They offer some possible explanations including differential access to labour markets, poor integration of general and mental health services and a lack of support for informal carers. Zinkler and Priebe [5] propose in relation to civil commitment rates that the values and beliefs of mental health professionals might be more important determinants than criteria stipulated in legislation. Concerning rates of involuntary detention, a recent study also implicated a country’s GDP, expenditure on healthcare and rates of absolute poverty [8].

In forensic care, similar arguments are given. Differences in legal frameworks, changes to legislation, clinical and legal thresholds for involuntary admission, diagnostic practices, public attitudes, deinstitutionalization, reinstitutionalization, transinstitutionalization, resource allocation and macroeconomic factors, increases in drug use, and the availability of professional training schemes in forensic mental health are given as possible reasons for differences between countries and within countries over time [1, 2, 10–12]. Dreßing and Salize [9] further highlight differences in data reporting practices across jurisdictions, and Zinkler and Priebe [5] that some observed differences might pertain to language and translation differences.

Chow et al. [13] conducted a qualitative study into experts’ opinions on what drives changes in institutionalized mental health care. Respondents’ proposed that a philosophy of deinstitutionalization, limited financial resources, limitations on the use of appropriate community services, and the prioritization of risk management have driven decreases in general psychiatric bed numbers and increases in forensic beds over time. The authors proposed that differences between countries might also be due to factors external to the profession. They offer a culture of risk aversion in the UK, the Psychiatrie-Enquête in Germany, and the Basaglia Law in Italy as examples.

## Aims and rationale

Much of the data described in the literature are outdated. Accordingly, what Salize and Dreßing [14] wrote 13 years ago still stands:

“…across Europe and all over the world there is a surprising shortage of basic information and evidence on the quantity and quality of services available for mentally disordered or ill offenders, the frequency of cases in specialized forensic facilities or the effectiveness of provided care in the various countries” (p. 336).

Furthermore, the literature is ambiguous as to possible explanations for heterogeneity in service provision. Accordingly, this paper aimed to update current knowledge on some key epidemiological indicators of forensic services in Europe. Experts from 17 countries were approached to gather data on total number of forensic cases, bed rates per 100,000, gender and average length of stay. This paper also investigated to what extent these indicators were associated with broader social indicators offered as possible explanations of the differences between countries as proposed in the literature. Specifically: GDP per capita (in current USD); percentage of GDP spent on health; prison population per 100,000; democracy index score; and general psychiatric bed rate per 100,000.

## Materials and methodology

A research action on long-term forensic psychiatric care was launched in 2013 in the framework of the EU-funded COST action IS1302 “Towards an EU research framework on Forensic Psychiatric care”. Experts in forensic mental health from nineteen EU countries participated in the COST action, which aimed to establish a basis for comparative evaluation, research on effective treatments and the development of ‘best practices’ in long-term forensic psychiatry in Europe. The present study was conducted between 2015 and 2017.

The study’s methodology was inspired by that of Salize and Dreßing [1]. A round table of COST action members was convened to discuss the structure of the questionnaire used in this study and generate a common definition of forensic services/patients. All experts in the COST action agreed on the focus of the questionnaire and related definitions.

### Data collection

The questionnaire comprised three parts: (1) preliminary information on forensic in-patients facilities provision and organization in the country; (2) number of forensic cases as in-patients in the country, psychiatric diagnosis, placement by kind of facility and mean length of stay of these patients in the last year; (3) and population of the country in the reference year; and source and quality of provided data. Comments about specific national characteristics could be added to the questionnaire. All participants answered the first part of the questionnaire on forensic in-patient facilities provision and organization in the country; these results are not shown here as the issue of service provision within forensic mental health inpatient services was the main object of a dedicated and published study in the framework of the same COST action and published elsewhere [2].

The questionnaire was completed by experts in forensic psychiatry participating in the COST action or who were closely associated with it. This network consisted of leading researchers and clinicians working in the field of forensic psychiatry. Each member had an interest in long-term care services and systems. Experts were only invited to participate if they had extensive experience working in secure settings and were familiar with the policies and services in their country. Experts were approached through professional networks, including the Forensic Section of the European Psychiatric Association (EPA), and the Long-Term Forensic Psychiatric Care Special Interest Group of the International Association of Forensic Mental Health Services (IAFMHS).

These questionnaires were returned to the authors of the questionnaire who cross-checked and harmonized data as far as possible. The sources used for the following variables: GDP per capita (in current USD); percentage of GDP spent on health; prison population per 100,000; democracy index score; and general psychiatric bed rates per 100,000 are described in Table 3.

### Data analysis

Epidemiological data are presented graphically. Associations between average length of stay and forensic bed prevalence rates, and wider social variables were investigated with Spearman’s RHO. SPSS version 24 was used [15]. Bootstrapping with 1000 samples was conducted given the small sample size [16]. Reported 95% confidence intervals are bootstrapped. Significance levels were set to *p* < 0.05.

### Definitions

Mentally disordered offenders whose mental disorder was related to their crime have diminished or absent criminal responsibility, and were placed in forensic facilities as in-patients were the population of interest. As the concept of criminal responsibility is not universally applicable and the admission criteria to forensic services differ widely among states, it was agreed that remanded (pre-trial) or sentenced prisoners transferred to a forensic hospital/unit and patients detained for treatment for a mental disorder under civil mental health law in forensic/secure hospitals should also be included in the survey. All experts contributed to this common definition of forensic psychiatric inpatient, length of stay and long-stay forensic psychiatric patients while a subgroup of researchers developed a written questionnaire which was reviewed and approved by all the participants to the action (Supplementary material).

## Results

Data were collected from the following 17 countries: Belgium, Germany, Latvia, Italy, Ireland, Poland, Portugal, The Netherlands, England & Wales, Scotland, Slovenia, Spain, Finland, France, Croatia, Macedonia and Lithuania. Data refer to 2013, the most recent year in which more complete information was available at the time of the survey. Where responses were not completed by a participating country, these countries are not presented in the analysis.

The annual number of in-patient cases in forensic psychiatric care across the various countries is a most basic epidemiological indicator. Table 1 shows data provided by experts that have used to calculate this rate. Most of the population statistics are census data collected at the end of 2013.

**Table 1:** Total number of forensic cases as in-patients in 2013 across countries participating in the survey

Country	Number of forensic psychiatric in patients	Population in 2013 (from National Statistical Offices)
Belgium	1,939	11,099,554
Germany^a^	5,752^a^ (Baden-Württemberg and Bavaria not included)	57,531,941 (Baden-Württemberg and Bavaria not included)
Latvia^a^	83 (Riga district only)	643,615 (Riga district only)
Italy	1,015	60,782,668
Ireland	91	4,593,100
Poland	2,200	38,495,000
Portugal	251	10,427,301
Netherlands	4,016 (1,858 TBS)	16,779,575
England & Wales	6,680	56,948,229
Scotland	522	5,327,700
Slovenia	42	2,060,663
Spain	666	46,727,890
Finland^a^	551	5,451,270
Croatia^a^	266	4,279,256
Macedonia	163	2,065,769
Lithuania	104	2,971,905

Notes: Germany: point prevalence, end of the year. Data source: State Ministries of Health. Baden-Württemberg and Bavaria not included

Latvia: point prevalence, end of the year. Data source: Statistical Office of Riga Centre of Psychiatry and Narcology

Italy: point prevalence, 01/06/2013. Data source: National survey, Italian Institute of Health

Ireland: point prevalence, 09/09/2013. Data source: Patient Register

Poland: point prevalence, end of the year. Data source: Institute of Psychiatry and Neurology in Warsaw Department of Health Care Organization

Portugal: point prevalence, end of the year. Data source: Portuguese Ministry of Justice

Netherlands: number of beds. Notes—TBS: 1858 patients; other forensic in-patient care: 1577; 581 beds/cells for prisoners in specialized prison wards/centre for prison mental health care. Number of beds in TBS-facilities may serve as an estimate for point prevalence. Data source: Pompe Foundation

England & Wales: point-prevalence, end of the year. Data source: NHS England

Scotland: point-prevalence, 26/11/2013. Data Source: Forensic Network Census

Slovenia: point-prevalence, 01/01/2014. Data Source: Patient Register

Spain: point-prevalence, end of the year. Data source: Menisterio del Interior, Spanish Government; Justice Department, Generalitat de Catalunya

Finland: point-prevalence, end of the year. Statistics limited to Helsinki University Hospital

Croatia: point-prevalence, end of the year. Data Source: University Department of Forensic Sciences, University of Split from 2 main psychiatric institutions hosting 80% of the whole forensic in-patient population

Macedonia: point prevalence, 01/01/2013. Data source: patient registers

Lithuania: point prevalence, referred to 31/12/2013, accessed on 31/12/2015. Data source: archival data of discharged patients

^a^Subnational data. Belgium: point prevalence, end of the year. Data source: service public fédéral Santé SPF Santé: Coordination fédérale en santé mentale. Service Public Fédéral Justice. Direction Générale administration pénitentiaire, Service Psycho-social Prison

Participating experts provided a subjective assessment of the quality of the data provided. This is shown in Table 2.

**Table 2:** Expert respondents’ assessment of data quality

Country	Data quality self-evaluation
Belgium	Sufficient/poor
Germany	Sufficient
Latvia	Sufficient
Italy	Sufficient
Ireland	Good
Poland	Sufficient
Portugal	Poor
Netherlands	Good
England and Wales	Good
Scotland	Good
Slovenia	Good
Spain	Good
Finland	Sufficient/poor
Croatia	Sufficient
Macedonia	Good
Lithuania	Sufficient/poor

**Fig. 1:**
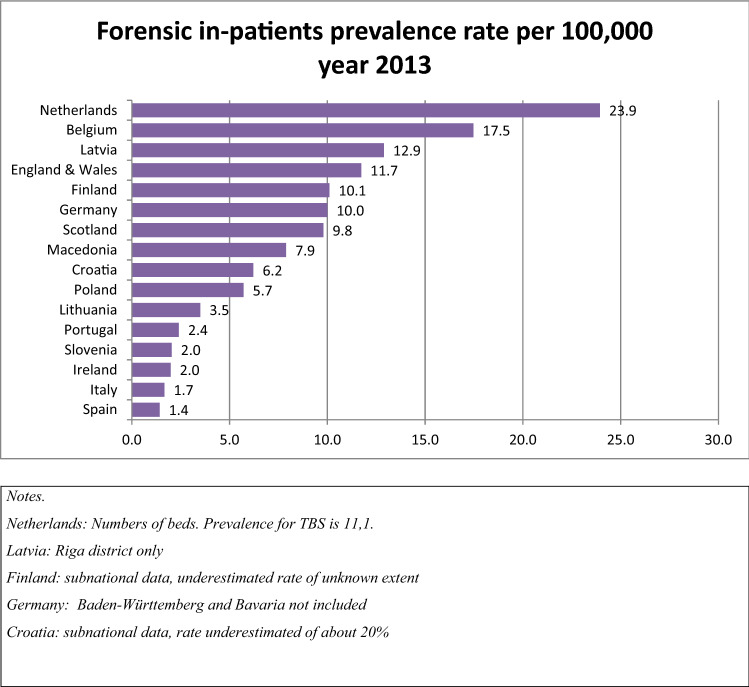
Forensic in-patient prevalence rate per 100,000 year 2013

Figure 1 displays the forensic in-patient prevalence rate per 100,000 across participating EU member states for the year 2013. The data clearly demonstrate a wide variation in the prevalence of forensic beds. The Netherlands has the highest number (23.9) and Spain the lowest (1.4), suggesting a 17-fold difference.

**Fig. 2:**
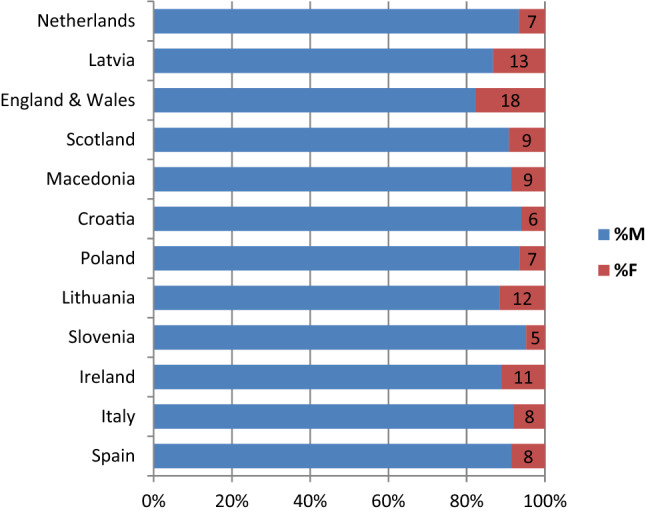
Gender of persons in forensic care as inpatients, year 2013

Figure 2 illustrates the proportion of male and female patients across participating states. Female patients are a clear minority in all forensic services. The state with the fewest female patients is Slovenia (5%) and the most is England and Wales (18%), with over three times the female population.

**Fig. 3:**
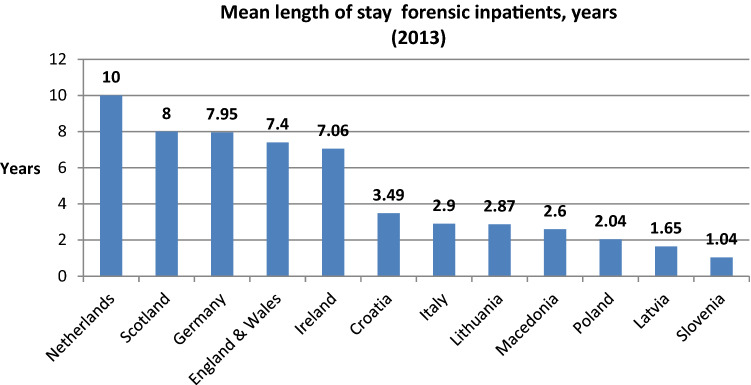
Mean length of stay for forensic in-patients (in years)

Figure 3 depicts the mean length of stay for forensic patients across the 12 states for which data were sent. There is a bipolar distribution. Seven states exhibit mean scores under 3.5 years with the remaining states averaging over 7 years. Slovenia’s mean length of stay (1.04 years) is approximately one-tenth that of the Netherlands (10 years).

There were no significant correlations between forensic bed rates per 100,000 and any of the social context variables. Average length of stay in years correlated significantly with GDP per capita (rho = 0.74, *p* = 0.006), percentage of GDP spent on healthcare (rho = 0.81, *p* = 0.002), and democracy index scores (rho = 0.70, *p* = 0.001). However, the interpretation of these results must be cautious given wide bootstrapped 95% confidence intervals and small sample. These results are depicted in Table 3.

**Table 3:** Spearman's RHO correlations between average length of stay, forensic bed rates per 100,000 and wider social variables

	GDP^a^	% GDP on healthcare^b^	Democracy index^c^	Prison population^d^	General psychiatric bed rates^e^	Forensic bed rates
LoS	00.736^**^	0.806^**^	0.697^*^	− 0.266	0.035	0.424
95% CI	0.278, 0.937	0.416, 0.964	0.112, 0.949	− 0.926, 0.484	− 0.750, 0.673	− 0.292, 0.864
Forensic bed rates	0.049	0.175	0.162	0.088	0.572	0.424
95% CI	− 0.671, 0.745	− 0.552, 0.760	− 0.584, 0.769	− 0.627, 0.761	− 0.089, 0.927	− 0.292, 0.864

Spearman’s RHO; *N* = 12; *p > 0.05; **p > 0.01; 95% CI results based on 1000 bootstrap samples

*LoS* length of stay

^a^Source: The World Bank Data Bank. GDP per capita in current USD. Accessed August 2019. Data for the year 2013. Single figure for England and Wales and Scotland representing United Kingdom

^b^Source: The World Bank Data Bank. Current Health Expenditure (% of GDP). Accessed September 2019. Data for the year 2013. Single figure for England and Wales and Scotland representing United Kingdom. Figure for Croatia from 2011, source: World Bank (2013). "World Development Indicators 2013." Washington, D.C.: World Bank

^c^Source: The Economist Intelligence Unit (2014). Democracy index 2013: Democracy in limbo. Data for the year 2013. Single figure for England and Wales and Scotland representing United Kingdom

^d^Source: EUROSTAT Prison capacity and number of persons held. Figures per 100,000. Data for the year 2013. Figure for North Macedonia from 2012, source: Aebi, M.F. & Delgrande, N. (2014). SPACE I: Council of Europe Annual Penal Statistics: Prison populations. Survey 2012. Strasbourg: Council of Europe

^e^Source: WHO European Health Information Gateway. Data for the year 2013. Figures per 100,000. Definitions of beds found: https://gateway.euro.who.int/en/indicators/hfa_488-5070-psychiatric-hospital-beds-per-100-000/. Figure for Netherlands from 2011, source: OECD Health Statistics 2013. Figure for Netherlands includes social care sector beds that might not be counted as psychiatric beds in other countries

## Discussion

This study collected data on key epidemiological indicators of forensic mental health services in 17 EU member states. Experts in forensic psychiatry from these states completed a questionnaire and rated the quality of available data on these indicators. Data were not available for all indicators across all states; this posed a challenge when comparing countries. However, the results suggest that there continues to be wide variation in forensic service provision. Average length of stay was approximately ten times greater in the Netherlands than Slovenia. In England and Wales, 18% of patients were female compared to 5% in Slovenia. There was a 17-fold difference in bed rates per 100,000 between the Netherlands and Spain. Experts assessed the quality of available data from ‘poor’ to ‘good’.

The literature has suggested that variation in the provision of forensic services might be associated with legal, cultural and economic factors [1, 3, 13]. A suitably powered statistical model with longitudinal data was beyond the scope of the present paper. However, very exploratory correlations with bootstrapping indicated that there were positive relationships between average length of stay and the wider social variables: GDP per capita, percentage of GDP spent on healthcare and democracy index scores.

Little can be confidently extracted from these associations given the small sample size (*N* = 12) but other literature has demonstrated similar results. For example, countries with a higher GDP and that spent more of this on healthcare had a longer average length of stay. This might reflect the proposition of Salize and Dreßing [1] who argued that there was a difference in service provision between countries in the Northern and Southern Europe. An inspection of Fig. 3 supports this.

A recent study explored differences in mental health service provision between Finland (Northern Europe) and Spain (Southern Europe) [7]. The authors reported a large difference in residential service use, with Finnish users engaging these services more often. This difference they attributed in part to familial structures and cultural differences, including the higher number of individuals living alone in Finland and the bonding and caring functions families were more likely to provide in Spain.

A second study found small associations between involuntary hospitalization rates and GDP per capita purchasing power parity and health-care spending per capita in mental health services across 24 countries [8]. These authors also found small associations with rates of foreign-born individuals in the population, total psychiatric bed numbers and absolute poverty. However, the absence of a significant association between GDP and forensic beds rates in the present study obfuscates the applicability of these findings to data in this project.

Further research is needed to explore the relationship between democracy scores and average length of stay. In the present study, GDP per capita correlated almost perfectly with the democracy index score variable (rho = 0.97, *p* < 0.001). See supplementary materials. It is not possible to meaningfully disentangle the association between democracy scores and average length of stay from the limited data in our study. Criminological literature suggests there is a strong correlation between higher trust and perceived legitimacy in governments, the amount spent on social welfare nationally, and incarceration rates [17]. Assessing the relationships between length of stay, forensic bed rates and these indicators may also be informative.

The present study and wider literature reveal variations in both service provision and the availability and quality of basic epidemiological data across countries [14]. Given the increase in forensic bed rates in recent years [3, 10, 11] and recent literature projecting an increase in the use of mental health services globally due to the social, ecological and economic consequences of the climate crisis [18–20], the need for systematically collected and published data with harmonized reporting standards across forensic services grows ever more important.

## Future research

Future research should adopt similar methods to Chow and Priebe [3]. These should combine data on key epidemiological indicators since 1990 across all countries for which there exists relevant and useable data. Appropriate measures of wider social context variables such as prison populations, GDP per capita, percentage of GDP spent of healthcare, democracy scores, attitudes towards mental illness and criminal justice, rates of absolute poverty, rates of employment and indicators on the structure of families should be collected. Analyses of variance between groups on relevant legal variables should be conducted. For instance, do key epidemiological variables differ significantly in states that (1) provide secure care for civil and forensic patients from those that provide care only for forensic patients, (2) offer forensic out-patient care and those that do not, or (3) those that offer specialized forensic mental health training/educational programmes and those that do not. A table describing these and similar distinctions as given in Salize and Dressing [14]: 232 would be a healthy starting point for these analyses.

Another key question from this study is how can countries best learn from each other to share best practices despite such diversity in service provision? International conferences play a role here, but more focused efforts are needed. Several groups of European experts have formed to tackle this, including the COST Action for which this project was conducted. A second is the Ghent Group. This group meets to discuss cross-border efforts to promote training, education and professional development for forensic psychiatrists [21]. Comparative studies into aspects of forensic services have great utility in identifying what works well and what does not; these can provide ‘roadmaps’ for coalescing around best practices. Two examples of this compare multi-agency working [22], and offender–patient pathways [23] in several jurisdictions. Finally, research should evaluate the potential for supranational trainee programmes for practitioners across Europe accredited by bodies like the European Psychiatric Association, the European Federation of Psychiatric Trainees, or the European Union of Medical Specialists [21].

## Limitations

A limitation of this study is the small sample size. This renders measures of association as exploratory. Participating experts’ ratings of data quality ranged from ‘poor’ to ‘good’ suggesting that all conclusions drawn must be viewed critically. Not all data used to measure wider social context variables were taken from the same year or source. Italy has closed forensic psychiatric hospitals and converted to fully residential services in 2017 with a 40% reduction in the number of patients compared to the numbers included in our analysis [24]. In Latvia and Finland, forensic epidemiological data were taken from specific districts only. In Germany, data are missing for Baden-Württemberg and Bavaria. However, the wider social context variables were nationally reported, which further emphasizes the exploratory nature of the investigated associations and highlights the need for more complete data. Although forensic epidemiological data for Scotland and England and Wales were collected separately, several of the wider social context variables were reported on a U.K. level (see Table 3). Despite these limitations, the data presented in this study represent the most recent overview of key epidemiological data in forensic services across 17 EU states.

## Conclusion

The present study found substantial differences on key forensic psychiatric epidemiological indicators between 17 EU member states. Reasons put forward for differences in mental health provision in the literature include broad social, political and economic differences. More specific explanations offered include a country’s GDP and the percentage of this spent on healthcare, the relationship between that of prison places and psychiatric beds, cultures of risk containment, familial and community support structures, levels of absolute poverty and legal frameworks directing involuntary and forensic treatment. Further research is needed to add clarity to the reasons for and consequences of this variation. Countries need to develop common definitions and recording practices and contribute to a publicly available database of such epidemiological indicators to further this research programme. This is a precondition for equity in forensic mental health services across Europe.

## Electronic supplementary material

Below is the link to the electronic supplementary material.

Supplementary file1 (XLSX 20 kb)

Supplementary file2 (DOCX 26 kb)

Supplementary file3 (DOCX 46 kb)
