# Supplementary material for: Forensic mental health in Europe: some key figures
Source: Soc Psychiatry Psychiatr Epidemiol. 2020 Jul 10;56(1):109–17. doi: 10.1007/s00127-020-01909-6 (PMC7847441; doi:10.1007/s00127-020-01909-6)
Supplement: Supplementary file 2 — Supplementary file2 (DOCX 26 kb) [file 127_2020_1909_MOESM2_ESM.docx]

| **Correlations** | | | | | | | | | | | |
| --- | --- | --- | --- | --- | --- | --- | --- | --- | --- | --- | --- |
|  | | | | | gdp | bed_rate | LoS_ave | democ | prison_pop | gen_psych_bed | prc_gdp_health |
| Spearman's rho | gdp | Correlation Coefficient | | | 1.000 | .049 | .736^**^ | .974^**^ | -.494 | -.102 | .888^**^ |
|  |  | Sig. (2-tailed) | | | . | .879 | .006 | .000 | .103 | .753 | .000 |
|  |  | N | | | 12 | 12 | 12 | 12 | 12 | 12 | 12 |
|  |  | Bootstrap^c^ | Bias | | .000 | -.027 | -.042 | -.017 | .031 | .009 | -.037 |
|  |  |  | Std. Error | | .000 | .366 | .162 | .038 | .225 | .382 | .111 |
|  |  |  | 95% Confidence Interval | Lower | 1.000 | -.671 | .278 | .864 | -.847 | -.801 | .565 |
|  |  |  |  | Upper | 1.000 | .745 | .937 | 1.000 | .058 | .693 | .993 |
|  | bed_rate | Correlation Coefficient | | | .049 | 1.000 | .424 | .162 | .088 | .572 | .175 |
|  |  | Sig. (2-tailed) | | | .879 | . | .170 | .614 | .787 | .052 | .585 |
|  |  | N | | | 12 | 12 | 12 | 12 | 12 | 12 | 12 |
|  |  | Bootstrap^c^ | Bias | | -.027 | .000 | -.041 | -.028 | -.001 | -.028 | -.029 |
|  |  |  | Std. Error | | .366 | .000 | .306 | .356 | .354 | .272 | .346 |
|  |  |  | 95% Confidence Interval | Lower | -.671 | 1.000 | -.292 | -.584 | -.627 | -.089 | -.552 |
|  |  |  |  | Upper | .745 | 1.000 | .864 | .769 | .761 | .927 | .760 |
|  | LoS_ave | Correlation Coefficient | | | .736^**^ | .424 | 1.000 | .697^*^ | -.266 | .035 | .806^**^ |
|  |  | Sig. (2-tailed) | | | .006 | .170 | . | .012 | .404 | .914 | .002 |
|  |  | N | | | 12 | 12 | 12 | 12 | 12 | 12 | 12 |
|  |  | Bootstrap^c^ | Bias | | -.042 | -.041 | .000 | -.041 | -.018 | -.004 | -.038 |
|  |  |  | Std. Error | | .162 | .306 | .000 | .206 | .360 | .368 | .139 |
|  |  |  | 95% Confidence Interval | Lower | .278 | -.292 | 1.000 | .112 | -.926 | -.750 | .416 |
|  |  |  |  | Upper | .937 | .864 | 1.000 | .949 | .484 | .673 | .964 |
|  | democ | Correlation Coefficient | | | .974^**^ | .162 | .697^*^ | 1.000 | -.465 | -.028 | .840^**^ |
|  |  | Sig. (2-tailed) | | | .000 | .614 | .012 | . | .128 | .931 | .001 |
|  |  | N | | | 12 | 12 | 12 | 12 | 12 | 12 | 12 |
|  |  | Bootstrap^c^ | Bias | | -.017 | -.028 | -.041 | .000 | .028 | .008 | -.038 |
|  |  |  | Std. Error | | .038 | .356 | .206 | .000 | .242 | .364 | .125 |
|  |  |  | 95% Confidence Interval | Lower | .864 | -.584 | .112 | 1.000 | -.842 | -.728 | .519 |
|  |  |  |  | Upper | 1.000 | .769 | .949 | 1.000 | .147 | .680 | .978 |
|  | prison_pop | Correlation Coefficient | | | -.494 | .088 | -.266 | -.465 | 1.000 | -.116 | -.690^*^ |
|  |  | Sig. (2-tailed) | | | .103 | .787 | .404 | .128 | . | .721 | .013 |
|  |  | N | | | 12 | 12 | 12 | 12 | 12 | 12 | 12 |
|  |  | Bootstrap^c^ | Bias | | .031 | -.001 | -.018 | .028 | .000 | .011 | .028 |
|  |  |  | Std. Error | | .225 | .354 | .360 | .242 | .000 | .352 | .221 |
|  |  |  | 95% Confidence Interval | Lower | -.847 | -.627 | -.926 | -.842 | 1.000 | -.737 | -.957 |
|  |  |  |  | Upper | .058 | .761 | .484 | .147 | 1.000 | .660 | -.095 |
|  | gen_psych_bed | Correlation Coefficient | | | -.102 | .572 | .035 | -.028 | -.116 | 1.000 | -.060 |
|  |  | Sig. (2-tailed) | | | .753 | .052 | .914 | .931 | .721 | . | .854 |
|  |  | N | | | 12 | 12 | 12 | 12 | 12 | 12 | 12 |
|  |  | Bootstrap^c^ | Bias | | .009 | -.028 | -.004 | .008 | .011 | .000 | -.024 |
|  |  |  | Std. Error | | .382 | .272 | .368 | .364 | .352 | .000 | .406 |
|  |  |  | 95% Confidence Interval | Lower | -.801 | -.089 | -.750 | -.728 | -.737 | 1.000 | -.904 |
|  |  |  |  | Upper | .693 | .927 | .673 | .680 | .660 | 1.000 | .582 |
|  | prc_gdp_health | Correlation Coefficient | | | .888^**^ | .175 | .806^**^ | .840^**^ | -.690^*^ | -.060 | 1.000 |
|  |  | Sig. (2-tailed) | | | .000 | .585 | .002 | .001 | .013 | .854 | . |
|  |  | N | | | 12 | 12 | 12 | 12 | 12 | 12 | 12 |
|  |  | Bootstrap^c^ | Bias | | -.037 | -.029 | -.038 | -.038 | .028 | -.024 | .000 |
|  |  |  | Std. Error | | .111 | .346 | .139 | .125 | .221 | .406 | .000 |
|  |  |  | 95% Confidence Interval | Lower | .565 | -.552 | .416 | .519 | -.957 | -.904 | 1.000 |
|  |  |  |  | Upper | .993 | .760 | .964 | .978 | -.095 | .582 | 1.000 |
| **. Correlation is significant at the 0.01 level (2-tailed). | | | | | | | | | | | |
| *. Correlation is significant at the 0.05 level (2-tailed). | | | | | | | | | | | |
| c. Unless otherwise noted, bootstrap results are based on 1000 bootstrap samples | | | | | | | | | | | |

Jack Tomlin, Ilaria Lega, Peter Braun, Harry G Kennedy, Vicente Tort Herrando, Ricardo Barroso, Luca Castelletti, Fiorino Mirabella, Franco Scarpa, Birgit Völlm and the experts of COST Action IS1302* Forensic Mental Health in Europe: Some Key Figures
