# Supplementary material for: Forensic mental health in Europe: some key figures
Source: Soc Psychiatry Psychiatr Epidemiol. 2020 Jul 10;56(1):109–17. doi: 10.1007/s00127-020-01909-6 (PMC7847441; doi:10.1007/s00127-020-01909-6)
Supplement: Supplementary file 3 — Supplementary file3 (DOCX 46 kb) [file 127_2020_1909_MOESM3_ESM.docx]

**Forensic in-patients prevalence across Europe**

The aim of this survey is to measure and compare the prevalence of the **“forensic psychiatric in-patients with needs for security and care”**” across the 19 Countries participating to the Action.

Mentally disordered offenders whose mental disorder is related to the crime, have diminished (or absent) criminal responsibility, and are placed in forensic facilities as in-patients are the focus of this survey.

As the concept of criminal responsibility is not universally applicable and the admission criteria to forensic services differ widely among States, please consider that: i) remanded (pre-trial) or sentenced prisoners transferred to forensic hospital/unit and, ii) patients detained for treatment for a mental disorder under civil mental health law in forensic/secure hospitals are also included in this survey.

**Please read the following definitions carefully before filling in the form**

**Forensic** = patients who need care and treatment in a secure setting.

A forensic patient is likely to mean:

1) a person detained for treatment for a mental disorder under criminal law mental health legislation in a forensic/secure psychiatric hospital

*and/or* and / or

2) a person detained for treatment for a mental disorder under civil mental health law in a forensic/secure psychiatric hospital

*and/or*

3) a person treated in a prison psychiatric hospital or unit

*and/or*

4) a person subject to compulsory or conditional treatment in the community arising from an

order of a criminal court.

**In-patients** = exclude out-patients, but include prisoners if they are in a prison psychiatric hospital/unit

**Psychiatric**= refers to mental disorder as locally defined for forensic admission, may include personality disorder, autism, addiction… if locally appropriate.

**Needs for care**= refers to criminogenic and psychiatric needs.

Long-stay forensic psychiatric patients, “who are not able to safely progress to a level of lower security due to internal and/or external factors”*,* will be a subgroup of the population identified through this survey.

**Length of stay** = it is the cross-sectional length of stay for all forensic patients on the census date.

Please consider that:

1) length of stay is since the date of admission to a psychiatric hospital, even if first admitted to another hospital or to a prison hospital, then transferred to the forensic hospital

2) mean with standard deviation, median, and sample size for estimating other parameters should be given.

**Please direct your questions on the form to**: ilaria.lega@iss.it

| ***Please fill in the form in the grey spaces, by following the instructions in italic.*** | | | | | |
| --- | --- | --- | --- | --- | --- |
|  |  | |  |  |  |
| **Country** |  | |  | **Date** |  |
|  |  | |  | |  |
| **Contact information:** | | | | | |
| *Please fill in the table below with the requested information* | | | | | |
| Contact person: | |  | | | |
| Position: | |  | | | |
| Organization: | |  | | | |
| Phone: | |  | | | |
| Fax: | |  | | | |
| Email: | |  | | | |
| Postal address: | |  | | | |

1. **Preliminary information**

**1.**

| **Forensic psychiatric patient definition** | |
| --- | --- |
| *Indicate with a yes or no whether or not the following statements are appropriate for your Country* | |
| Yes  No | To be considered a forensic psychiatric patient a person needs to have committed a crime |
| Yes  No | A connection between the criminal behaviour and the mental disorder is necessary to be committed to a forensic psychiatric institution. |
| Yes  No | It is necessary for the offender to have diminished (or absent) criminal responsibility to be admitted to a forensic psychiatric institution. |
| Notes: | |

**2.**

| **Kind of in-patients facilities for forensic psychiatric patients**  *Select below the kind of in-patient facility where you can find forensic psychiatric patients (as defined on page 1) in your Country. Multiple choice allowed* |
| --- |
| Forensic hospital  Forensic ward(s) in psychiatric hospitals  Forensic departments or wards within general hospitals  Prison psychiatric hospitals or wards  Others |
| *If “Others” please describe here:* |

**3.**

| **Exclusion criteria for forensic admission by psychiatric diagnosis**  *Indicate with a yes or no whether or not the following statement is appropriate for your Country* | |
| --- | --- |
| Yes  No | Some psychiatric disorders are excluded from forensic psychiatric care |
| *If “Yes”, please specify here:* | |

**4.**

| **Specialization by mental disorders and/or patients groups**  *Indicate with a yes or no whether or not the following statement is appropriate for your Country* | |
| --- | --- |
| YesNo | In-patients services for forensic psychiatric patients with specific mental disorders are available |
| *If “Yes” please specify for which disorder and/or patients groups:* | |

**5.**

| **Specialization by level of structural security and/or procedures**  *Indicate with a yes or no whether or not the following statements are appropriate for your Country* | |
| --- | --- |
| Yes  No | High, medium, low secure in-patients facilities for forensic psychiatric patients are available |
| Yes  No | Long stay in-patients facilities for forensic psychiatric patients are available |
|  | *If applicable, please specify long stay definition in your Country:* |
| Notes: | |

1. **Number of Forensic Cases as in-patient in 2013**

*Please fill in the tables below with the requested information referring to year 2013.*

*Use point prevalence or census data on 31st December 2013 if available.*

**6.**

| Data Source: |  |
| --- | --- |
| Collected on: | *Specify as following (Day/Month/2013) the day on which data that you are using was collected* |
| Evaluation of data quality: | *Based on validity, reliability, integrity and completeness*  *please indicate the quality of data available to you*  Good quality  Sufficient  Poor quality |
| Notes: | |

**7.**

| Total Number of forensic psychiatric  in-patients: |  | N=  Male N= Female N= |
| --- | --- | --- |
| Notes: | | |

**8.**

| Number of forensic psychiatric in-patients  by main psychiatric diagnosis: | Schizophrenia | N=  Male N= Female N= |
| --- | --- | --- |
|  | Other psychotic disorders | **N=**  **Male N= Female N=** |
|  | Personality disorders | **N=**  **Male N= Female N=** |
|  | Substance-related and addictive  disorders | **N=**  **Male N= Female N=** |
|  | Affective and anxiety disorders | **N=**  **Male N= Female N=** |
|  | Others diagnosis | **N=**  **Male N= Female N=** |
| Notes: | | |

**9.**

| Number of forensic psychiatric in-patients  by legal status: | Pre-trial | N=  Male N= Female N= |
| --- | --- | --- |
|  | Post-trial | **N=**  **Male N= Female N=** |
|  | Detained for treatment for a mental disorder under **criminal law**  **mental health legislation**  (if applicable) | **N=**  **Male N= Female N=** |
|  | Detained for treatment for a mental disorder under **civil mental health law**  (if applicable) | **N=**  **Male N= Female N=** |
|  | Others | **N=**  **Male N= Female N=** |
| Notes: | | |

**10.**

| Number of forensic psychiatric in-patients  by kind of facility: | In forensic hospital | N=  Male N= Female N= |
| --- | --- | --- |
|  | In forensic ward(s) in psychiatric hospitals | **N=**  **Male N= Female N=** |
|  | In forensic departments or wards within general hospitals | **N=**  **Male N= Female N=** |
|  | In prison psychiatric hospitals or wards | **N=**  **Male N= Female N=** |
|  | Others | **N=**  **Male N= Female N=** |
| Notes: | | |

**11.**

| Number of forensic psychiatric in-patients  by service specialization: | Patients admitted to services  for specific mental disorders or  patients groups | N=  Male N= Female N= |
| --- | --- | --- |
|  | Patients in high secure | **N=**  **Male N= Female N=** |
|  | Patients in medium secure | **N=**  **Male N= Female N=** |
|  | Patients in low secure | **N=**  **Male N= Female N=** |
|  | Patients in long stay | **N=**  **Male N= Female N=** |
| Notes: | | |

**12.**

| Total Number of patients first-ever admitted to a forensic psychiatric  in-patient facility in 2013: |  | N=  Male N= Female N= |
| --- | --- | --- |
| Notes: | | |

**13.**

| Total Number of patients discharged from forensic psychiatric in-patient facilities in 2013: |  | N=  Male N= Female N= |
| --- | --- | --- |
| Notes: | | |

**14.**

| Mean length of stay in forensic psychiatric in-patients facilities of the population identified in table 7 page 4 | Mean (years) |  |
| --- | --- | --- |
|  | Standard deviation |  |
|  | Sample size for estimating mean |  |
|  | Median |  |
| Notes: | | |

**C. Population of your Country in 2013**

**15.**

| *Please fill in the table below with the requested information.* | |
| --- | --- |
| Population of your Country in 2013: | *Indicate the number of inhabitants of your Country in 2013*  N= |
| Age limits for admission to forensic psychiatric services: |  |
| Population of your Country above age limits for forensic admission in 2013: | *Please indicate the number of inhabitants of your Country above the age limits for forensic admission in 2013*  N= |
| Data Source: | |

1. **Number of Forensic Cases as in-patient in 2014**

*Please fill in the tables below with the requested information referring to year 2014. Use point prevalence or census data on 31st December 2014 if available.*

**16.**

| Data Source: |  |
| --- | --- |
| Collected on: | *Specify as following (Day/Month/2014) the day on which data that you are using was collected* |
| Evaluation of data quality: | *Based on validity, reliability, integrity and completeness*  *please indicate the quality of data available to you*  Good quality  Sufficient  Poor quality |
| Notes: | |

**17.**

| Total Number of forensic psychiatric  in-patients: |  | N=  Male N= Female N= |
| --- | --- | --- |
| Notes: | | |

**18.**

| Number of forensic  psychiatric in-patients  by psychiatric diagnosis: | Schizophrenia | N=  Male N= Female N= |
| --- | --- | --- |
|  | Other psychotic disorders | **N=**  **Male N= Female N=** |
|  | Personality disorders | **N=**  **Male N= Female N=** |
|  | Substance-related and addictive  disorders | **N=**  **Male N= Female N=** |
|  | Affective and anxiety disorders | **N=**  **Male N= Female N=** |
|  | Others diagnosis | **N=**  **Male N= Female N=** |
| Notes: | | |

**19.**

| Number of forensic  psychiatric in-patients  by legal status | Pre-trial | N=  Male N= Female N= |
| --- | --- | --- |
|  | Post-trial | **N=**  **Male N= Female N=** |
|  | Detained for treatment for a mental disorder under **criminal law**  **mental health legislation** | **N=**  **Male N= Female N=** |
|  | Detained for treatment for a mental disorder under **civil mental health law** | **N=**  **Male N= Female N=** |
|  | Others | **N=**  **Male N= Female N=** |
| Notes: | | |

**20.**

| Number of forensic psychiatric in-patients  by kind of facility | In forensic hospital | N=  Male N= Female N= |
| --- | --- | --- |
|  | In forensic ward(s) in psychiatric hospitals | **N=**  **Male N= Female N=** |
|  | In forensic departments or wards within general hospitals | **N=**  **Male N= Female N=** |
|  | In prison psychiatric hospitals or wards | **N=**  **Male N= Female N=** |
|  | Others | **N=**  **Male N= Female N=** |
| Notes: | | |

**21.**

| Number of forensic psychiatric  in-patients  by service specialization | Patients admitted to services for specific mental disorders or patients groups | N=  Male N= Female N= |
| --- | --- | --- |
|  | Patients in high secure | **N=**  **Male N= Female N=** |
|  | Patients in medium secure | **N=**  **Male N= Female N=** |
|  | Patients in low secure | **N=**  **Male N= Female N=** |
|  | Patients in long stay | **N=**  **Male N= Female N=** |
| Notes: | | |

**22.**

| Total Number of patients first-ever admitted to a forensic psychiatric  in-patient facility in 2014 |  | N=  Male N= Female N= |
| --- | --- | --- |
| Notes: | | |

**23.**

| Total Number of patients discharged from forensic psychiatric in-patient facilities in 2014 |  | N=  Male N= Female N= |
| --- | --- | --- |
| Notes: | | |

**24.**

| Mean length of stay in forensic psychiatric in-patients facilities of the population identified in table 17 page 7 | Mean (years) |  |
| --- | --- | --- |
|  | Standard deviation |  |
|  | Sample size for estimating mean |  |
|  | Median |  |
| Notes: | | |

1. **Population of your Country in 2014**

**25.**

| *Please fill in the table below with the requested information.* | |
| --- | --- |
| Population of your Country in 2014 | *Indicate the number of inhabitants of your Country in 2014*  N= |
| Population of your Country above age limits for forensic admission in 2014 | *Please indicate the number of inhabitants of your Country above the age limits for forensic admission in 2014*  N= |
| Data Source: |  |

**THE COMPLETED FORM SHALL BE SENT TO** [**ilaria.lega@iss.it**](mailto:ilaria.lega@iss.it)
